# Unusual Effect of α-olefins as Chain Transfer Agents in Ethylene Polymerization over the Catalyst with Nonsymmetrical Bis(imino)pyridine Complex of Fe(II) and Modified Methylalumoxane (MMAO) Cocatalyst

**DOI:** 10.3390/ijms232214384

**Published:** 2022-11-19

**Authors:** Nina V. Semikolenova, Mikhail A. Matsko, Vladimir A. Zakharov, Wen-Hua Sun

**Affiliations:** 1 Boreskov Institute of Catalysis, Pr. Lavrentieva 5, 630090 Novosibirsk, Russia; 2 Key Laboratory of Engineering Plastics, Beijing National Laboratory for Molecular Sciences, Institute of Chemistry, Chinese Academy of Sciences, Beijing 100190, China

**Keywords:** bis(imino)pyridyl complex of Fe(II), ethylene polymerization, polyethylene, molecular weight, molecular weight distribution, α-olefins

## Abstract

Ethylene polymerization with bis(imino)pyridlyiron precatalysts generally produces linear polyethylene (PE) even with the presence of α-olefins because α-olefins are not incorporated into polymeric products. Interestingly, α-olefins, such as hexene-1 or butene-1, have been found to act as effective chain transfer agents in the ethylene polymerization promoted by nonsymmetrical bis(imino)pyridyliron complexes with modified methylalumoxane (MMAO), resulting in higher catalytic activities with higher amounts of polymers with lower molecular weights, and, more importantly, narrower molecular weight distributions of the resultant polyethylenes (PE). This phenomenon confirms the assistance of α-olefins in the chain-termination reaction of iron-initiated polymerization and regeneration of the active species for further polymerization. Besides higher activities of the catalytic system, the formation of linear PE with trans-vinylene terminal groups and lower molecular weights are explained. The observation will provide a new pathway for enhancing catalytic activity and improving the quality of polyethylenes obtained by regulation of molecular weights and molecular weight distribution.

## 1. Introduction

Ethylene polymerization by bis(imino)pyridyliron complexes with the cocatalyst methylalumoxane (MAO) was first reported in 1998 [1,2,3]. These catalytic systems were highly active, with activities comparable to the most effective metallocene catalysts. Subsequently, modified bis(imino)pyridine derivatives controlled the catalytic performances of their iron complexes in affecting activities and molecular weights of the obtained high-density polyethylene [4,5,6,7,8,9,10,11] and even lower-molecular-weight products, such as oligomers (α-olefins) [12,13,14,15,16,17,18]. Extensive investigations have provided various modifications of the ligands and tuned the catalytic activities of their metal complexes and controlled structural features of the resultant polyethylenes (and oligomers) along with understanding the reaction mechanism; this progress has been revealed in a number of review articles [19,20,21,22]. As the copolymerization was considered, the trials of ethylene polymerization by the iron precatalysts were explored in the presence of α-olefins [19,23], indicating that no traces of copolymers were confirmed.

In addition, another drawback of the iron-based catalysts is their low thermo-stability in polymerization, commonly leading to deactivation at temperatures above 40 °C. To overcome such a restriction, an efficient solution has been developed by using bulk substituents of bis(imino)pyridine derivatives and the formation of iron complexes with increased catalytic activities at elevated temperatures [24,25,26,27,28]. The catalytic system of 2-[1-(2,6-dibenzhydryl-4-chlorophenylimino)ethyl]-6-[1-mesityliminoethyl]pyridy liron dichloride with the cocatalyst MMAO was conducted for ethylene polymerization in the temperature range of 65–80 °C and produced polyethylenes with high molecular masses (270–290 kg/mol) and relatively broad molecular mass distributions (M_w_/M_n_ = 9.1–15) [29].

It has been a challenge to obtain advanced polyolefin through the copolymerization of ethylene with α-olefins; therefore, it is necessary to explore the influence of α-olefins on ethylene polymerization based on the bis(imino)pyridine iron complexes. The possibility to control molecular weight, molecular weight distribution, the content of branching and branching distribution in the case of copolymerization of ethylene with α-olefins is the most important issue for the production of different grades of polyethylene. Therefore, it is important to obtain more experimental data in this area for a new, promising catalytic system based on the bis(imino)pyridine iron complexes. The copolymerization of ethylene with α-olefins is the usual pathway to control the content of branching in polyethylene, but there are no literature data on the effect of α-olefins on the molecular weight and MWD of polyethylene produced over catalysts with bis(imino)pyridine iron complexes.

The extensive research of ethylene polymerization in the presence of α-olefins [23] with nonsymmetrical bis(imino)pyridine iron complexes is revisited. We have found some unusual catalytic performances, including higher activities and lower molecular weights of resultant polyethylenes as well as narrower molecular weight distributions. This phenomenon is interpreted with the assistance of α-olefins in the chain-transfer reaction of iron-initiated polymerization and regeneration of active species. Herein, the new observation and possible explanation are described in detail.

## 2. Results and Discussion

1. The peculiarities of ethylene polymerization kinetics and molecular mass characteristics of polyethylene, produced by the catalytic system composed of nonsymmetrical bis(imino)pyridine Fe(II) complex (*LFeCl_2_) and cocatalyst MMAO.

**Figure 1:**
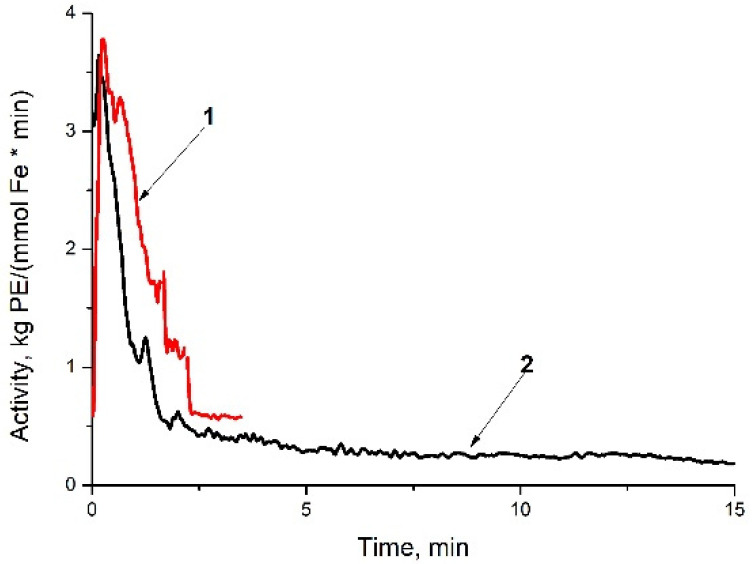
Kinetic curves of ethylene polymerization for exps. 1 and 2 in Table of Figure 2.

**Figure 2:**
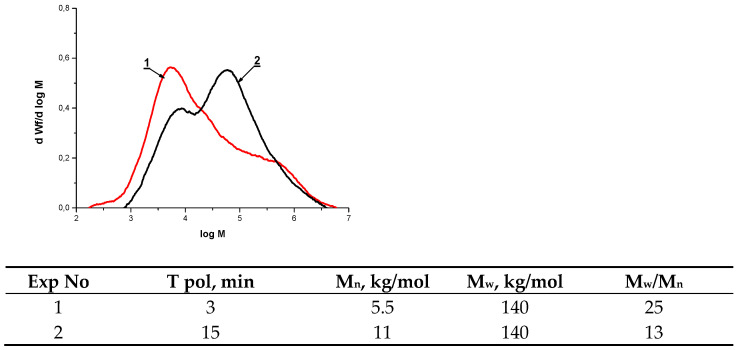
MWD curves of PE samples obtained from different polymerization durations: curve 1—3 min, curve 2—15 min.

As noted earlier in the literature [25,26,27,28,29,30], the catalytic system including *LFeCl_2_ and cocatalyst MMAO possesses enhanced thermal stability and gives the possibility to obtain polyethylene (PE) in a high yield at a polymerization temperature of 65 °C. However, high PE yield is achieved due to the extremely high activity of the catalyst in the initial period of the reaction (less than 2 min); then, the polymerization rate sharply decreases and becomes a stationary but rather low level (Figure 1, curve 1). The extension of polymerization duration from 3 to 15 min results in drastic changes in PE molecular weight distribution (Figure 2). The polymer obtained at a short polymerization time (3 min) has a broad MWD (the value M_w_/M_n_ = 25) with a primary contribution from low-molecular-weight components with molecular masses lower than 10 kg/mol (Figure 2, curve 1).

The polymer obtained in 15 min was also characterized by broad MWD (with a value of M_w_/M_n_ = 13), but in this sample, the fraction with a molecular mass higher than 50 kg/mol prevailed (Figure 2, curve 2).

It should be mentioned that, according to the data [29], in the ethylene polymerization with the catalyst *LFeCl_2_/MMAO at 65 °C, the chain termination reaction via transfer to aluminum alkyls (i.e., predominantly to MeAl(i-Bu)_2_), attended in the cocatalyst MMAO), is predominant. In this case, as was noted [29], with the increase in PE yield, a decrease in the content of the chain-transfer agent (MeAl(i-Bu)_2_)) occurs, resulting in the formation of PE with high molecular mass due to the increase in the content of the high-molecular-mass fraction, as can be seen from the comparison of MWD curves shown in Figure 2.

At the same time, we cannot exclude the presence of two groups of active centers in this catalytic system producing polymers with different molecular masses. In this case, if the partial deactivation of the active centers, observed in the initial stage of polymerization (Figure 1, curve 1), predominantly embraces the active centers that produce low-molecular-mass PE, the contribution of the high-molecular-mass fraction should increase with the extension of polymerization time (Figure 2).

**Figure 3:**
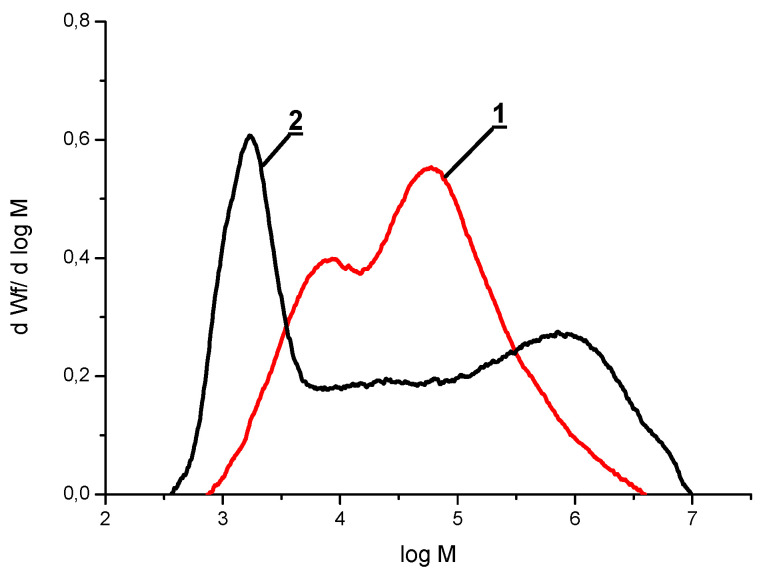
MWD curves for polymer samples produced in exps. 1 and 2 in Table 1.

**Figure 4:**
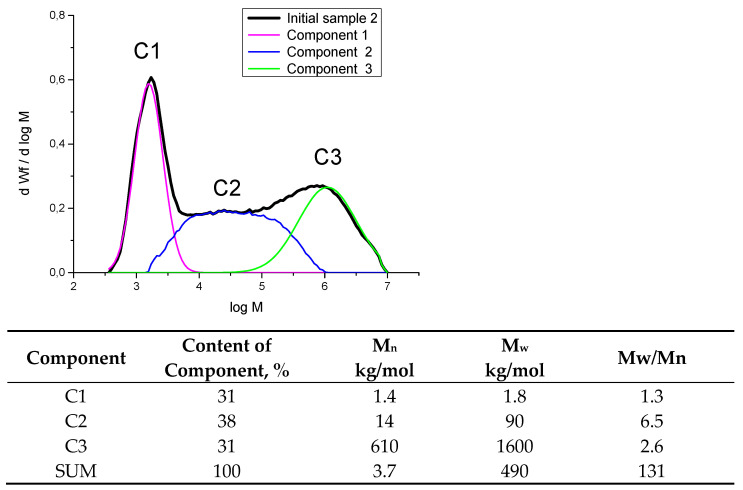
Data on the deconvolution of MWD curve for PE sample obtained from polymerization over the catalyst *LFeCl_2_/MMAO with TMA added into polymerization (exp. 2 in Figure 3).

Data obtained in the polymerization run over this catalytic system using AlMe_3_ as the additional chain-transfer agent (Table 1, Figure 3 and Figure 4) argue for the assumption of the presence of two groups of active centers, producing polymers with different molecular masses in the catalytic system *LFeCl_2_/MMAO.

It is seen that the introduction of AlMe_3_ into the reaction medium (exp. 2 in Table 1) results in drastic changes in PE molecular weight distribution, with the formation of a polymer with very broad bimodal MWD (Figure 3, curve 2). The observed strong broadening of MWD (the value M_w_/M_n_ = 138) occurs due to the formation of two PE fractions with sharply different molecular masses: a low-molecular-mass fraction with MM in the region of 1 kg/mol and a high-molecular-mass fraction with MM in the region of 1000 kg/mol. We believe that the data shown in Figure 3 and Table 1 indicate the presence of several types of active centers that have different reactivity in the chain-transfer reaction with AlMe_3_.

For a more precise estimation of molecular weights and the content of separate components constituting a complicated MWD curve, shown in Figure 3, we have conducted a theoretical deconvolution of the MWD curve on some individual components. The obtained results are presented in Figure 3 and the table in the footnotes of this figure. The obtained data allow us to propose the presence of three groups of active centers with different levels of reactivity in the chain-transfer reaction with AlMe_3_. In particular, the system contains the centers with very high reactivity in the chain-transfer reaction with AlMe_3_, which produce PE with a very low molecular mass (component 1, the value of M_w_ = 1.8 kg/mol with narrow MWD, the value of M_w_/M_n_ ~ 2). At the same time, the active centers, which produce PE with a very high molecular mass (component 3, the value of M_w_ = 1600 kg/mol with narrow MWD, the value of M_w_/M_n_ = 2.6), are practically inactive in the chain-transfer reaction with AlMe_3_.

Thus, it is possible to assume that the multisite character of the catalytic system *LFeCl_2_/MMAO depends on the polymerization conditions and appears in different reaction conditions, particularly with the addition of some compounds that can participate in the chain-transfer reactions.

2. Ethylene polymerization over the catalyst *LFeCl_2_/MMAO in the presence of α-olefins.

It was noted in the Introduction that one of the peculiarities of the catalysts based on bis(imino)pyridyl complexes of Fe(II) is their low copolymerization ability at the ethylene polymerization in the presence of α-olefins [21,25]. There are no literature data on the copolymerization ability of the nonsymmetric bis(imino)pyridyl complex *LFeCl_2_, considered in this work. Taking into account the strong influence of the bis(imino)pyridyl ligand structure and composition on the ethylene polymerization over the corresponding iron complexes and molecular mass characteristics of the resultant polymer, we have studied the effect of α-olefins on the kinetics and molecular mass characteristics of the produced polymers for the system *LFeCl_2_/MMAO.

**Figure 5:**
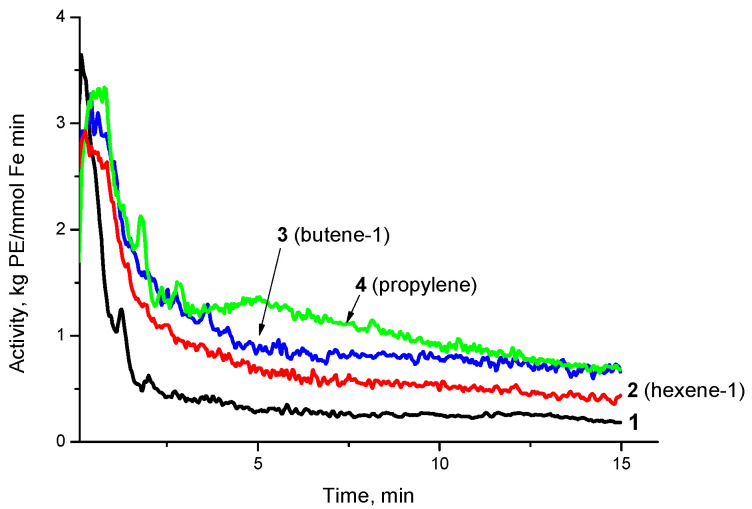
Kinetic curves of ethylene polymerization for the experiments presented in Table 2 (numbers of the curves correspond to the experiment numbers in Table 2).

Table 2 and Figure 5 represent data on the effect of α-olefins (hexene-1, butene-1 and propylene) upon polymer yield, the polymerization rate and the shape of the kinetic curves.

It can be seen that, in the ethylene polymerizations in the presence of α-olefins, the value of the PE yield increases. In all cases, the catalyst shows very high initial activity, and the increase in PE yield can be attributed to a less sharp decrease in the catalyst activity with polymerization time and a higher level of stationary activity during 2–15 min. (Figure 5).

**Figure 6:**
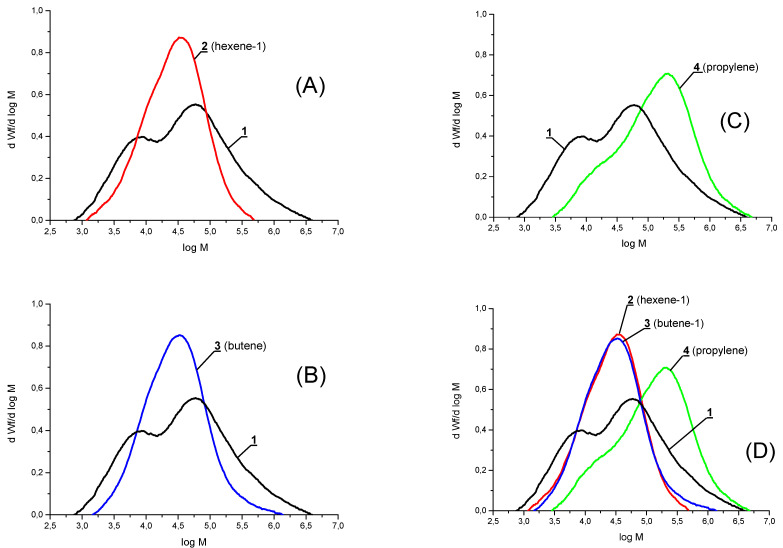
MWD curves for polymer samples obtained during ethylene polymerization in the presence of hexene-1 (**A**, curve 2), butene-1 (**B**, curve 3), propylene (**C**, curve 4) and in the absence of α-olefins (curve 1 in **A**–**D**); (**D**)—MWD curves for all polymers obtained in exps. 1–4 (Table 3).

Data on the effect of α-olefins on the molecular structure of polymers, obtained in the ethylene polymerization in the presence of hexene-1, butene-1 and propylene (molecular mass and polydispersity of polymers (M_w_/M_n_ value) and the content of terminal groups with various compositions and melting temperatures) are summarized in Table 3 and Figure 6.

**Figure 7:**
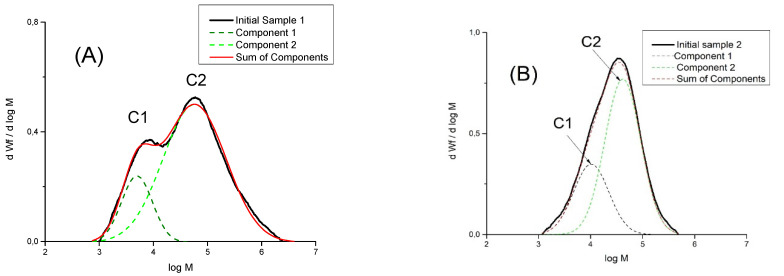
Data on the deconvolution of MWD curves of PE samples 1 and 2 obtained from ethylene polymerization in the absence of α-olefins (**A**) and from ethylene polymerization with hexene-1 (**B**).

The data of Table 3 (exps. 2 and 3) and Figure 6 show that the presence of hexene-1 and butene-1 in ethylene polymerization leads to drastic changes in the molecular mass characteristics of the produced polymers in comparison with those obtained in homopolymerization (exp. 1). The PE samples of exps. 2 and 3 show narrower monomodal MWD (the value M_w_/M_n_ = 3.0–3.4). It is seen that the decrease in the M_w_ value in exps. 2 and 3 mainly originates from the decrease in the contribution of the high-molecular-mass component present in the bimodal “homopolymer” (exp. 1 in Figure 6A,B). We suppose that these results can be explained by the additional reaction of the chain transfer with these comonomers taking place in the ethylene polymerization. For a more precise estimation of the effect of α-olefin on the molecular weight of separate components of bimodal polyethylene obtained in polymerization in the absence of α-olefin, we have made the theoretical deconvolution of MWD curves, represented in Figure 6A (curves 1 and 2), into two components. The results obtained by deconvolution are shown in Figure 7A,B.

Data presented in Figure 7 and Table 4 show that the addition of hexene-1 into ethylene polymerization leads mainly to a decrease in the M_w_ value for the high-molecular-mass part C2 for sample 2 in comparison with sample 1 (from 130 kg/mol to 56 kg/mol). This means that active centers producing high-molecular-mass components of bimodal polyethylene are involved mainly in the chain transfer reaction with hexene-1.

**Figure 8:**
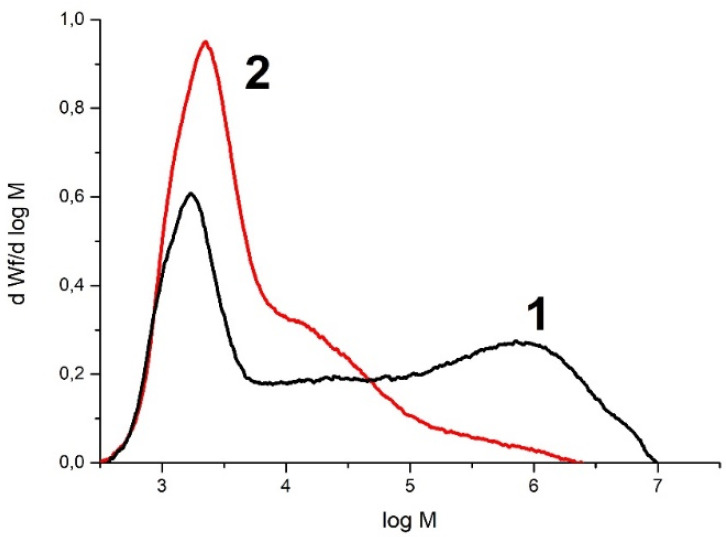
MWD curves for polymers obtained from ethylene polymerization over catalytic system *LFeCl_2_/MMAO + TMA in the absence of hexene-1 (curve 1) and in the presence of hexene-1 (curve 2) (for data on the polymerization, see Table 5).

It was shown above (Table 1 and Figure 3) that in the case of ethylene polymerization on the catalytic system *LFeCl_2_/MMAO + AlMe_3_, polyethylene with a very broad MWD is formed (M_w_/M_n_ = 138). The MWD curve of this polymer contains two separate components with very low molecular weight (M_w_ = 1.8. kg/mol) and very high molecular weight (M_w_ = 1600 kg/mol) formed probably on the different active sites (Figure 4). Data on the ethylene polymerization in the presence of hexene-1 over the catalytic system *LFeCl_2_/MMAO + AlMe_3_ and the molecular weight distribution of the polymer produced are presented in Figure 8 and Table 5. It is seen that the addition of hexene-1 into polymerization leads to a great narrowing of MWD (value of M_w_/M_n_ decreases from 138 to 12.7) because of the strong decrease in the value of M_w_ from 470 kg/mol to 33 kg/mol and the very small deviation in the value of M_n_ (3.4–2.6 kg/mol). This means that active sites producing the high-molecular-weight part in the initial bimodal polyethylene (sample 1 in Table 4) are very effective in the transfer reaction of the polymer chain with hexene-1, contrary to the active sites producing low-molecular-weight parts in the bimodal polyethylene.

Data on the molecular mass characteristics of the polymer obtained in the polymerization run in the presence of propylene (exp. 4 in Table 3 and Figure 6C) distinctly differ from that of the runs with hexene-1 and butene-1. The introduction of propylene results in an increase in PE molecular mass. Though the MWD curve retains its bimodal mode, the deposit of the low-molecular-mass fraction substantially decreases, resulting in a noticeable decrease in PE polydispersity (the value of M_w_/M_n_ decreases from 13 to 6.3). Therefore, unlike hexene-1 and butene-1, propylene does not participate in the chain-transfer reaction; moreover, it distinctly reduces the rate of the chain-transfer reaction with MeAl(i-Bu)_2_, which, according to the data [29], prevails in the ethylene polymerization with the catalyst *LFeCl_2_/MMAO.

**Figure 9:**
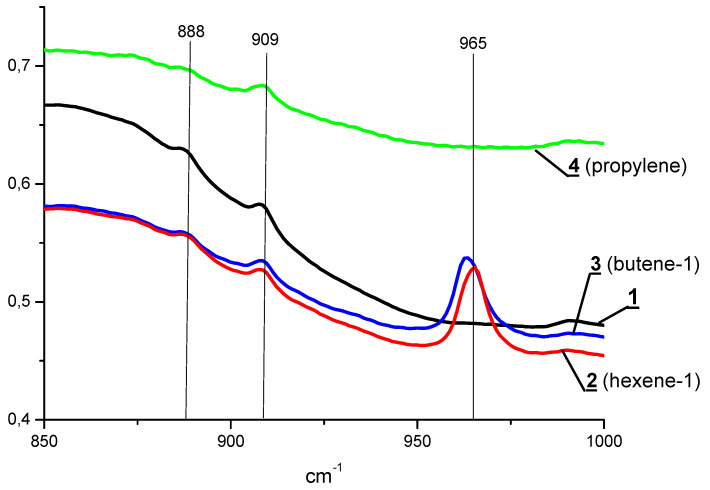
IR spectra of polymers, presented in Table 3. (Numbers of the spectra correspond to the experiment numbers in Table 3).

At the establishment of the reactions that determine the produced PE molecular mass, it is necessary to take into consideration data on the composition and the number of terminal groups, the formation of which is governed by the mechanism of the chain-transfer reactions. In particular, the polymer obtained in the ethylene polymerization over the catalyst *LFeCl_2_/MMAO (Table 3, exp. 1) contains mainly methyl terminal groups formed via the chain termination reaction with MeAl(i-Bu)_2_ [29]. In this polymer, the content of vinyl groups, arising due to the chain termination reaction with ethylene, is very low. We have found that in polymerization in the presence of hexene-1 and butene-1, a polymer containing a substantial number of terminal trans-vinylene groups is formed (Table 3, exps. 2 and 3 and Figure 9).

**Scheme 1:**
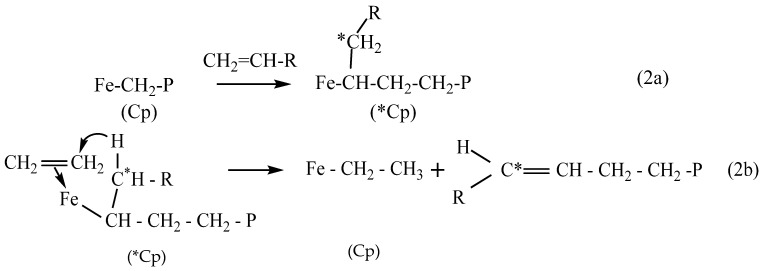
Chain transfer reaction with formation of trans-vinylene groups in polymer produced with *LFeCl_2_/MMAO catalyst during ethylene polymerization in the presence of hexene-1 and butene-1.

On the whole, the data on the decrease in the molecular mass of PE, obtained at the addition of hexene-1 and butene-1 into polymerization, the formation of trans-vinylene groups, not observed in the “homopolymer”, and the absence of branching in these polymers, allow us to suspect a new additional chain-transfer reaction, shown in Scheme 1 (reactions 2a and 2b), proceeding in this case.

As the first stage, this scheme includes the reaction of the 2,1-incorporation of α-olefin into the active center (C_p_) of the growing polymer chain (2a). It should be mentioned that the absence of the branches in the polymer formed during ethylene polymerization in the presence of hexene-1 and butene-1 demonstrates that in these conditions, the 1,2-incorporation of α-olefin into the growing polymer chain is practically negligible.

As the first stage (2a) this scheme includes the reaction of the 2,1-incorporation of α-olefin into the active center (C_p_) of the growing polymer chain. It should be mentioned that the absence of branches in the polymer during ethylene polymerization in the presence of hexene-1 and butene-1 demonstrates that, in these conditions, the 1,2-incorporation of α-olefin into the growing polymer chain is practically negligible.

In the second stage (2b), the β-hydride transfer from the secondary carbon atom *C, positioned in the alkyl branching of the *C_p_ structure, to the ethylene coordinated on the iron atom of the active center occurs. Then, the active center is regenerated, and a polymer containing a trans-vinylene group is formed. It should be noted that, probably, the rate of this reaction is much higher than that of the possible incorporation of coordinated ethylene into the Fe-CHR(P) bond in the C_p_* structure, which can result in the formation of branchings in the resultant PE.

**Scheme 2:**
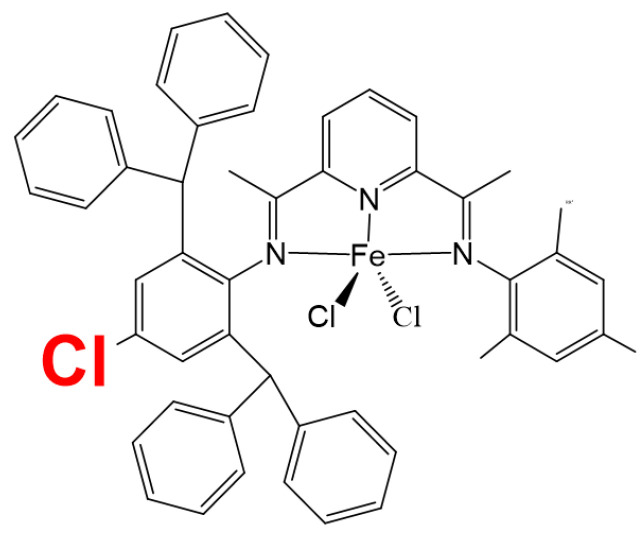
Structure of the iron complex (*LFeCl2) used in this work.

The data on the molecular mass characteristics of the polymer prepared from ethylene polymerization in the presence of propylene (Table 3, exp. 4 and Figure 6C) completely differs from those obtained from the addition of hexene-1 and butene-1. It is seen the addition of propylene leads to the formation of a polymer with a higher molecular weight and narrower MWD. Evidently, the reaction of chain transfer via Scheme 2 does not occur in the presence of propylene. The data presented in Figure 6C show that propylene interacts mainly with active sites, which produce low-molecular-weight polymers. This interaction takes place in the same way via the 2,1 incorporation of propylene into the growing polymer chain with the formation of the structure Fe-CH(CH_3_)-CH_2_-CH_2_P (C_p_**), but the reaction of β-hydride transfer from the methyl branching in the Cp** structure to the coordinated ethylene does not occur. Probably, in this case, the conversion of the structure (C_p_**) into the active center proceeds through the exchange of the alkyl ligands at its interaction with Me_2_Al(i-Bu)_2_. Therefore, the ethylene polymerization in the presence of propylene takes place mainly on active centers which produce high-molecular-weight polymer.

## 3. Materials and Methods

Commercial trimethylaluminum (AlMe_3_) was used as a heptane solution (1 M).

2-[1-(2,6-dibenzhydryl-4-chlorophenylimino)ethyl]-6-[1-mesityliminoethyl]pyridyl iron dichloride (*LFeCl_2_) (Scheme 2) was prepared according to a previously published procedure [30]. In the polymerization experiments, the iron complexes were used as solutions in CH_2_Cl_2_ (1 µmol Fe/mL).

All experiments were carried out in sealed high vacuum systems using “break seal” techniques.

### 3.1. Polymerization

Ethylene polymerization was performed in a 0.5 L steel reactor. A sealed glass ampoule with 0.5 mL of the solution of the iron complex in CH_2_Cl_2_ was placed into the reactor. The reactor was heated at 80 °C under vacuum for 1 h and cooled to 25 °C, then charged with heptane (200 mL) and the solution of cocatalyst MMAO in heptane or solution of MMAO + AlMe_3_. After setting up the desired polymerization temperature, ethylene pressure was inserted into the reactor. Hexene-1 (1.2 mol/L), butene-1 or propylene (1.0 mmol/L) were introduced into the reactor in one portion before starting the reaction. The reaction was started by breaking the ampoule with the iron complex. During the reaction, the stirring speed, the temperature and the ethylene pressure were maintained at a constant rate through the automatic computer-controlled system for the ethylene feed, recording the ethylene consumption and providing the kinetic curve output both as a table and as a graph. After a prescribed time, the reactor was vented, and the obtained solid product was separated and dried at ambient conditions to constant weight. The detailed experimental conditions are given below in the footnotes of the tables.

### 3.2. Measurements of PE Molecular Weight (MW) and Molecular Weight Distribution (MWD)

MWD measurements were performed using a high-temperature gel permeation chromatography (GPC) PL 220 system equipped with an RI detector in 1,2,4-trichlorobenzene stabilized with 0.0125 % BHT at a flow rate of 1 mL/min at 160 °C. The polymers were analyzed using a set of Olexis columns. Samples were dissolved at a concentration of 0.5–2.5 mg/mL depending on the molecular weights. Data collection and handling were carried out using Cirrus GPC Software and Multi Detector Software; data were collected at 1 point per second. The instrument was calibrated using polyethylene and polystyrene standards with narrow MWD in the range of 13,200,000–540 g/mol.

The MWD curve deconvolution technique is described elsewhere [31].

The content of methyl groups and C=C double bonds (vinyl a. b. 909 cm^−1^, vinylidene a. b. 888cm^−1^ and trans-vinylene a.b. 965 cm^−1^ groups) in PE samples has been determined by FTIR spectroscopy [32] using a Shimadzu FTIR 8400 S IR spectrometer.

## 4. Conclusions

It is found that the introduction of AlMe_3_ an additional chain transfer agent during ethylene polymerization over the catalytic system *LFeCl_2_ with the cocatalyst MMAO results in sharp changes in the molecular mass and molecular weight distribution of the produced polyethylene.

In the presence of AlMe_3_, bimodal polyethylene with a very broad polydispersity (M_w_/M_n_ = 138) is obtained due to the formation of a substantial amount of a low-molecular-mass polymer fraction with M_w_ = 1.8 kg/mol, which is practically absent in the polymer produced in the absence of AlMe_3_. The polymer prepared in the presence of AlMe_3_ contains as well as a high-molecular-weight component with M_w_ = 1600 kg/mol and M_w_/M_n_ = 2.6. The obtained results indicate that in the catalytic system *LFeCl_2_/MMAO, active centers with different reactivities in the chain transfer reaction with AlMe_3_ are present. In particular, this catalyst contains centers with a very high rate of chain transfer with AlMe_3_, producing a low-molecular-mass polymer fraction with M_w_ = 1.8 kg/mol. At the same time, the active centers that are practically inactive in the chain transfer reaction with AlMe_3_ are present, giving a polymer with a very high molecular mass (M_w_ = 1600 kg/mol) and narrow MMD (M_w_/M_n_ = 2.6).

During ethylene polymerization over the system *LFeCl_2_/MMAO in the presence of α-olefins (hexene-1, butene-1 and propylene), we have found that in all cases, the reaction of ethylene copolymerization with α-olefins does not proceed, and linear polyethylene (practically without branches) is formed. However, we have found the unusual effects of α-olefins on the polymerization kinetics (the shape of the kinetic curves and polymer yield) as well as on the molecular mass and molecular weight distribution of polyethylene. It was found that in the presence of hexene-1 and butene-1, the molecular mass of the produced polymer decreases, and a monomodal polyethylene with a narrower MMD is formed. These results argue that hexene-1 and butene-1 act as additional efficient chain transfer agents in ethylene polymerization over this catalytic system. The results of the comparative analysis of the MMD curves of the bimodal polyethylene formed in the absence of α-olefins and monomodal polymers obtained in the presence of hexene-1 and butene-1 allow us to propose that this new chain transfer reaction mainly occurs with the participation of the active centers, producing a high-molecular-mass component of bimodal polyethylene.

It was found that in the polymers obtained in the presence of hexene-1 and butene-1, a noticeable amount of trans-vinylene terminal groups (0.33 RCH = CHR’ per one polymer chain), absent in polymers formed without α-olefins, are formed.

Based on the obtained experimental results, the scheme of the new reaction of chain termination that occurs at the ethylene polymerization in the presence of α-olefin over the catalyst *LFeCl_2_/MMAO (Scheme 2) was proposed. This scheme includes two stages. In the first stage (2a), the 2,1-incorporation of α-olefin into the growing polymer chain, connected to the Fe ion in the active center, occurs. In the second stage (2b), the transfer reaction of the hydrogen atom bound to the β-carbon atom in the alkyl branching, formed after the 2,1-incorporation of α-olefin into the growing polymer chain, to the ethylene molecule coordinated on the iron atom of the active center proceeds. As a result of this reaction, a polymer containing a terminal trans-vinylene group and an Fe-CH_2_CH_3_ bond in the active center is formed. The proposed scheme of chain termination reaction in the presence of α-olefins provides an explanation for the absence of branching in the produced polymer (the copolymerization does not occur). Evidently, in the catalyst containing the *LFeCl_2_ complex, the reaction of the 2,1-incorporation of α-olefin into the growing polymer chain prevails in comparison with the reaction of the 1,2-incorporation, mainly proceeding at the ethylene copolymerization with α-olefins over all known catalytic systems.

The results obtained from ethylene polymerization in the presence of propylene distinctly differ from that with hexene-1 and butene-1. In this case, polyethylene with an increased molecular weight and a narrower MWD is formed. This polymer does not contain terminal trans-vinylene groups. Evidently, in this case, after the 2,1-incorporation of propylene into the growing polymer chain, the reaction of β-hydride transfer from the methyl branching does not occur. Moreover, the presence of methyl branching in the α-position of the growing polymer chain hinders the chain transfer reaction with MeAl(i-Bu)_2_, the main chain transfer agent in these conditions.

## Figures and Tables

**Table 1:** Data on the effect of the addition of AlMe_3_ into polymerization with the catalyst *LFeCl_2_/MMAO on the molecular weight and molecular weight distribution of produced PE.

Exp. No.	AlMe_3_	PE Yield ^1^, kg/mmol Fe	Activity, kg PE/ (mmol Fe×min)	M_n_ kg/mol	M_w_ kg/mol	M_w_/M_n_
1	-	10	0.67	11	140	13
2	^2^ AlMe_3_	6.4	0.43	3.4	470	138.0

^1^ Polymerization at 65 °C, ethylene pressure 5 bar, for 15 min, *LFeCl_2_—2.5 µmol/L, MMAO—5 mmol/L. ^2^ AlMe_3_ (5 mmol/L) was added into polymerization.

**Table 2:** Data on the effect of α-olefins on the polymer yield and polymerization rate for ethylene polymerization over the catalyst *LFeCl_2_/MMAO.

Exp. No.	α-olefin ^1^	PE Yield kg PE/mmol Fe	R_p_, kg PE/(mmol Fe×min)	
			^2^ R_p_^max^	^3^ R_p_^st^
1	-	10.0	3.5	0.2
2	hexene-1	11.6	2.9	0.6
3	butene-1	14.0	3.2	0.8
4	propylene	18.4	3.3	1.0

Polymerization conditions: Fe = 2.5 µmol/L, temperature 65 °C, ethylene pressure 5 bar, MMAO = 5 mmol/L for 15 min. ^1^ Concentration of hexene-1 1.2 mol/L, butene-1 and propylene 1 mol/L. ^2^ Initial (maximal) polymerization rate for 1 min. ^3^ Average polymerization rate for the more stable period of polymerization (5–15 min).

**Table 3:** Data on the effect of α-olefins on the molecular structure of polymers obtained from ethylene polymerization in the presence of α-olefins.

Exp. No. ^1^	α-olefin (mol/L)	M_n_ kg/mol	Pn	M_w_ kg/mol	M_w_/M_n_	CH_2_ = CHR		RCH = CHR’		-CH_3_		Tm ^2^, °C
						(1 *)	(2 *)	(1 *)	(2 *)	(1 *)	(2 *)	
1	-	11	393	140	13	0.04	0.03	-	-	1.5	1.2	134.9
2	hexene-1	15	535	45	3.0	0.05	0.06	0.3	0.32	1.1	1.2	134.3
3	butene-1	16	571	54	3.4	0.06	0.07	0.3	0.34	2.0	2.3	134.9
4	propylene	46	1642	290	6.3	0.04	0.12	-	-	0.6	2.0	135.5

^1 ^ Experiment numbers correspond to those in Table 2. (1 *)—content of groups per 1000 C; (2 *)—content of groups calculated per one polymer chain. ^2^ Melting temperature of polymers (DSC data).

**Table 4:** Data on molecular weights of separate components C1 and C2 calculated according to the results in Figure 7.

Sample ^1^	Component	Content of Component, %	M_peak_ kg/mol	M_n_ kg/mol	M_w_ kg/mol	M_w_/M_n_
1	C1	19	4.9	4.0	6.2	1.5
	C2	81	57.5	25	130	5.3
	SUM	100		12.5	107	8.5
2	C1	30	10	7.7	14	1.8
	C2	70	41.5	30	56	1.9
	SUM	100	35	16	43	2.7

^1^ Numbers correspond to those in Table 3.

**Table 5:** Comparative data on the ethylene polymerization over catalyst *LFeCl_2_/MMAO + TMA in the absence of hexene-1 (exp. 1) and in the presence of hexene-1 (exp. 2).

Exp. No.	PE Yield, kg/mol	Activity kg PE	M_n_, kg/mol	M_w_, kg/mol	M_w_/M_n_
		mmol Fe × min			
1	6.4	0.43	3.4	470	138
2 ^1^	9.6	0.64	2.6	33	12.7

Polymerization at 65 °C, ethylene pressure 5 bar, for 15 min. LFeCl_2_—2.5 μmol/L, MMAO—5 mmol/L, TMA—5 mol/L. ^1^ C_6_H_12_—1.2 mol/L.
